# Acute Upper Limb Ischemia: A Case of Successful Thrombosuction and Catheter-Directed Thrombolysis in a Resource-Limited Hospital

**DOI:** 10.1016/j.jaccas.2025.105724

**Published:** 2025-10-19

**Authors:** Abiseka P. Baskoro, Enrico A. Budiono, Heru Sulastomo, Hadiyana A. Hafiz

**Affiliations:** a Department of Cardiology and Vascular Medicine, Universitas Sebelas Maret, Surakarta, Indonesia; b Department of Cardiology and Vascular Medicine, Universitas Sebelas Maret Hospital, Sukoharjo, Indonesia; c Faculty of Medicine, Universitas Sebelas Maret, Surakarta, Indonesia

**Keywords:** acute limb ischemia, case report, resource-limited, upper ALI, vascular

## Abstract

**Background:**

Acute limb ischemia (ALI) involving the upper limbs is a vascular emergency that represents only 5% to 10% of all ALI cases. Prompt diagnosis and intervention are crucial to prevent irreversible damage, particularly in resource-limited hospitals.

**Case Summary:**

A 56-year-old woman hospitalized with atrial fibrillation and acute decompensated heart failure developed sudden left upper limb weakness and sensory loss. Bedside Doppler ultrasonography confirmed axillary artery occlusion (Rutherford IIb). Given limited resources, she underwent successful manual thrombosuction followed by catheter-directed thrombolysis with alteplase, restoring distal perfusion without complications.

**Discussion:**

This case illustrates a rare but critical presentation of cardioembolic upper limb ALI. Despite limited vascular technology, a pragmatic endovascular approach led to limb salvage. It highlights the feasibility of guideline-adapted care in nontertiary settings.

**Take-Home Message:**

Even in hospitals with limited facilities, early recognition and tailored endovascular strategies can yield successful outcomes in upper limb ALI.

## Graphical Abstract

**Figure:**
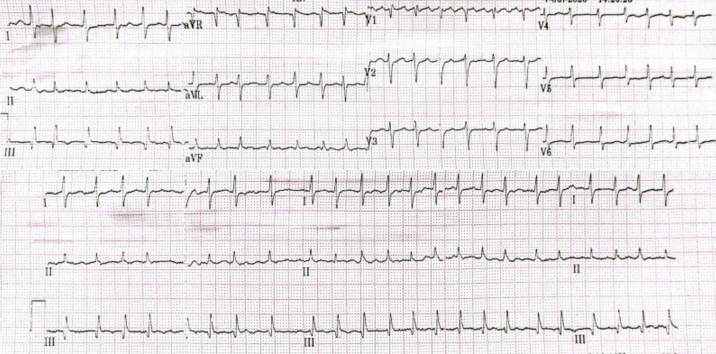

Acute limb ischemia (ALI) is a sudden decrease in limb perfusion that threatens tissue viability and requires urgent intervention.^1^ ALI more commonly affects the lower extremities, and involvement of the upper limbs accounts for <10% of all cases, making it a relatively rare clinical presentation.^2^ Despite its rarity, upper limb ALI carries a significant risk of morbidity, including permanent functional loss or limb amputation, if not addressed promptly.^1^^,^^3^ The condition demands immediate recognition and rapid revascularization to prevent irreversible ischemic injury.^3^ This poses a critical challenge in resource-limited settings, where advanced endovascular facilities and surgical options may not be readily available. Therefore, documenting successful management strategies, such as manual thrombosuction and catheter-directed thrombolysis (CDT), becomes essential to highlight feasible, life- and limb-saving interventions, even under constrained conditions in nontertiary hospitals.

**Take-Home Message**

• Even in hospitals with limited facilities, early recognition and tailored endovascular strategies can yield successful outcomes in acute upper limb ischemia.

## Case Illustration

**Figure 1:**
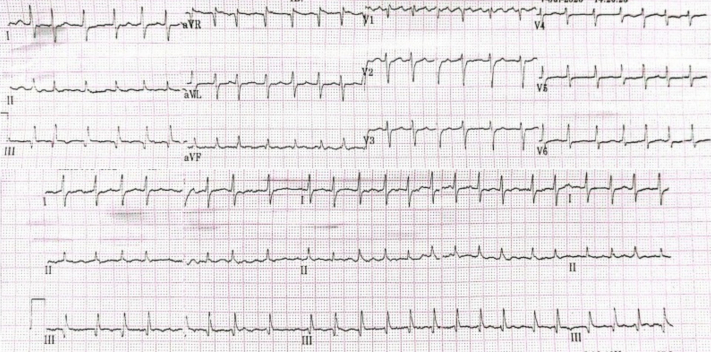
Baseline ECG Showing AF-RVR The 12-lead ECG on admission revealed AF-RVR at 140 beats/min and a normal QRS axis. This irregularly irregular rhythm and tachycardia were consistent with the patient's presentation of palpitations and dyspnea. AF-RVR = atrial fibrillation with rapid ventricular response; ECG = electrocardiogram.

**Figure 2:**
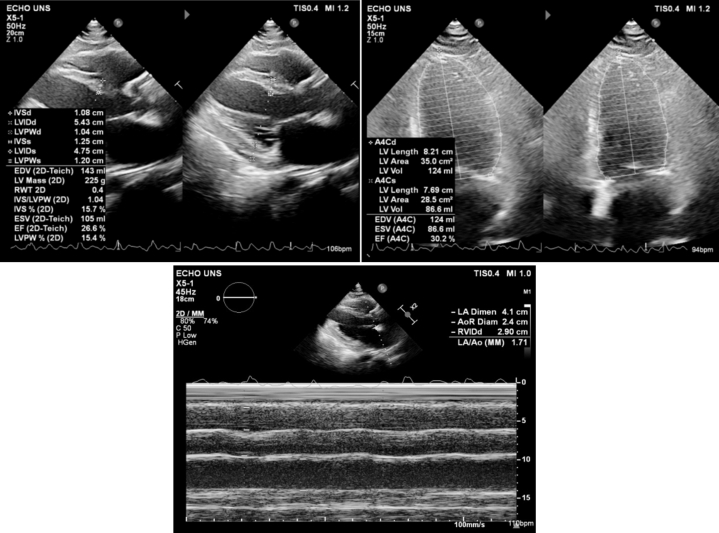
Findings on Transthoracic Echocardiography Echocardiographic assessment revealed left atrial dilatation, eccentric left ventricular hypertrophy, and segmental wall motion abnormalities. The left ventricular ejection fraction was reduced to 30.2%, and right ventricular function was impaired (TAPSE = 8 mm).

A 65-year-old woman presented with palpitations and shortness of breath. She had a history of poorly controlled hypertension but no other known comorbidities. She also reported intermittent claudication in her left hand over the past few months, described as mild, nonpainful episodes of transient numbness without significant functional limitation. On examination, she was hypertensive (blood pressure: 143/75 mm Hg) with an irregular heart rate of 148 beats/min and a noticeable pulse deficit. The electrocardiogram of the patient is shown in Figure 1. Transthoracic echocardiography revealed significant structural and functional abnormalities, including left atrial and ventricular dilation, eccentric left ventricular hypertrophy, segmental wall motion abnormalities, reduced left ventricular ejection fraction (30.2%), and right ventricular dysfunction (TAPSE: 8 mm). Echocardiographic findings are shown in Figure 2. The patient was diagnosed as having atrial fibrillation with rapid ventricular response and acute decompensated heart failure. She was hospitalized and was started on intravenous furosemide, guideline-directed medical therapy (GDMT) for heart failure, and anticoagulation with warfarin 2 mg. Her CHA_2_DS_2_-VA score was 3.

**Figure 3:**
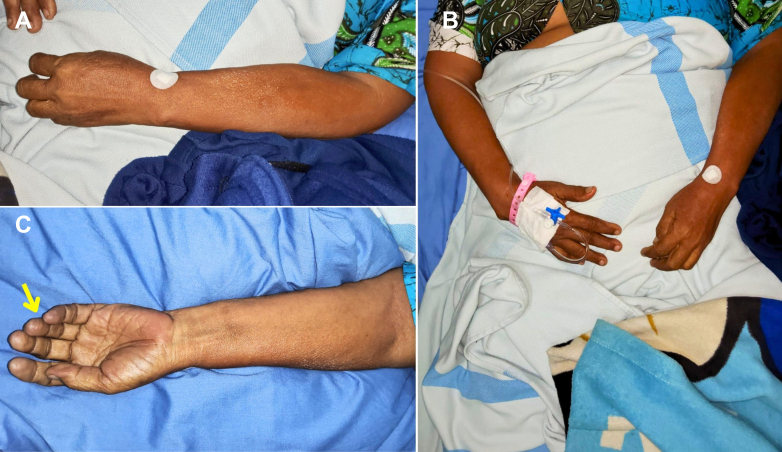
Clinical Presentation of Left Upper Limb Ischemia On hospitalization day 2, the patient developed acute ischemic symptoms in the left upper extremity, including pallor, coldness, and absent radial pulse. The hand appeared cyanotic with delayed capillary refill and absent digital oxygen saturation. These clinical signs suggested acute arterial occlusion, requiring urgent vascular evaluation. Yellow arrows indicate pallor of the affected limb, consistent with acute arterial occlusion requiring urgent vascular evaluation.

**Figure 4:**
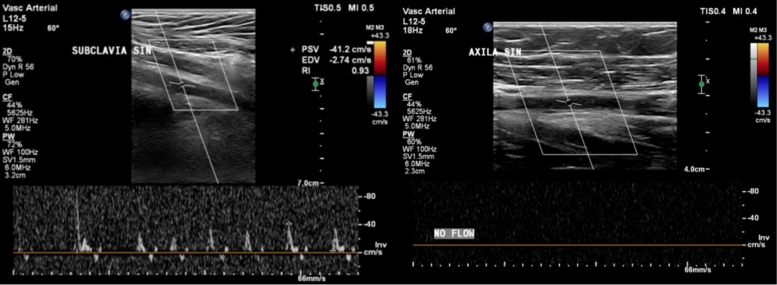
DUS Demonstrating Absence of Flow in Left Axillary Artery Bedside DUS showed an absence of flow in the left axillary artery, confirming arterial occlusion. This finding established the diagnosis of ALI classified as Rutherford grade IIb, indicating an immediate need for revascularization to prevent irreversible tissue damage. ALI = acute limb ischemia; DUS = Doppler ultrasound.

**Figure 5:**
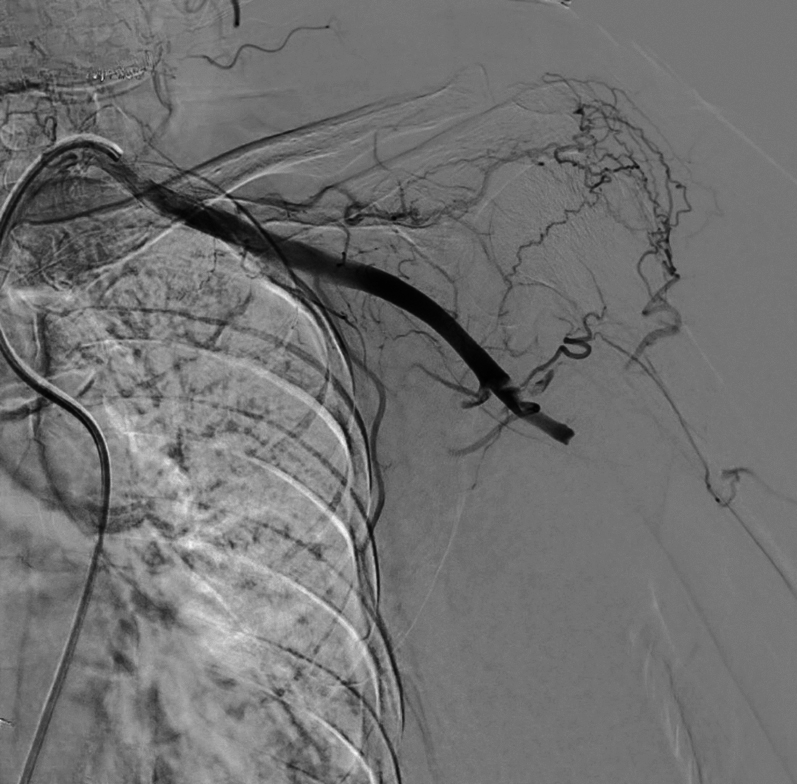
DSA Confirming Total Occlusion of Left Axillary Artery Diagnostic DSA showed abrupt cessation of contrast flow at the left axillary artery, consistent with total arterial occlusion. No perfusion was visualized distal to the blockage. The angiographic image confirmed the embolic nature of the occlusion and guided the decision for endovascular thrombosuction and thrombolysis. DSA = digital subtraction angiography.

On hospitalization day 2, despite improvement in atrial fibrillation and heart failure symptoms, the patient developed sudden pain, pallor, and numbness in the left upper limb, as shown in Figure 3. Physical examination revealed a cold, pale extremity with an absent radial pulse, capillary refill time exceeding 3 seconds, and undetectable oxygen saturation in the left fingers. Motor strength was reduced (Medical Research Council grade 3), and sensory loss extended up to the elbow. Emergent Doppler ultrasonography showed absent flow in the left axillary artery, consistent with Rutherford grade IIb upper limb ALI, as shown in Figure 4. Digital subtraction angiography (DSA) is shown in Figure 5.

**Figure 6:**
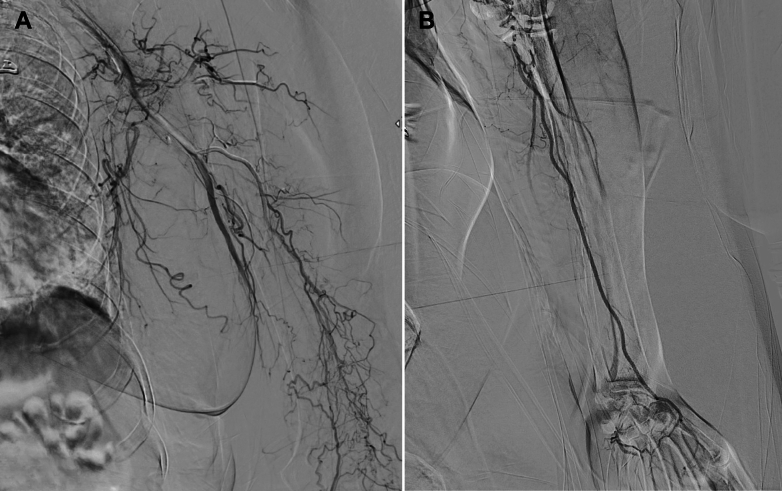
Postintervention Angiography Showing Revascularization of Left Upper Limb Arteries Follow-up DSA after manual thrombosuction and catheter-directed thrombolysis demonstrated restored perfusion through the radial artery and into the palmar arch. Mild residual thrombus was noted with slow flow in the ulnar artery, but overall vascular patency was achieved. This outcome signified successful limb salvage. DSA = digital subtraction angiography.

**Figure 7:**
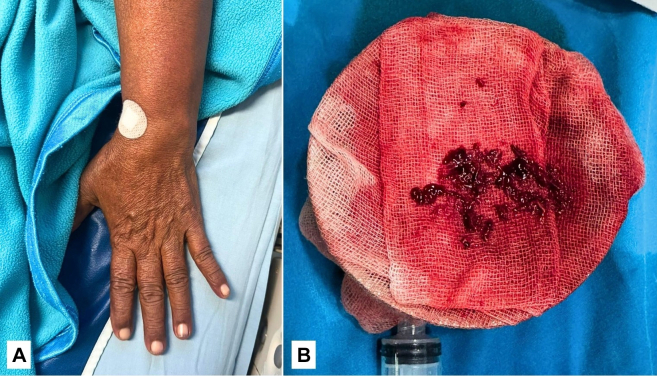
Clinical and Procedural Outcomes After Endovascular Intervention (A) The clinical appearance of the left upper limb showed improved color and capillary refill, with near-complete motor and sensory recovery. (B) Manual thrombosuction retrieved multiple thrombus fragments using a 6-F JR catheter and 10-mL syringe, despite the absence of advanced aspiration devices. These results highlight the feasibility of basic endovascular techniques in resource-limited settings.

The patient was immediately treated with intravenous heparin, followed by emergency manual thrombosuction using a 10-mL syringe and 6-F Judkins right catheter, and CDT with alteplase (3 mg bolus, followed by 1 mg/h infusion via syringe pump), along with continuous low-dose heparin (200 U via sheath). Postintervention DSA is demonstrated in Figure 6. Digital oxygen saturation improved to >90%, the limb appeared warm and well perfused, and radial and brachial pulses were palpable (Figure 7). Only mild residual numbness persisted, indicating successful revascularization. The patient was discharged on GDMT for heart failure, warfarin 4 mg, and atorvastatin 40 mg.

## Discussion

ALI is a vascular emergency and among the most urgent yet potentially reversible complications of peripheral artery disease.^3^ The majority of ALI cases involve the lower extremities given their larger vascular territory and higher atherosclerotic burden, with upper limb ALI accounting for only about 5% to 10% of all cases, making it an uncommon but clinically significant entity.^2^^,^^4^ Its rarity often contributes to diagnostic delays and management challenges, particularly in resource-limited settings where advanced vascular interventions may not be readily available.

In patients with atrial fibrillation, ALI typically arises from cardioembolic mechanisms.^5^, ^6^, ^7^ The chaotic atrial contractions lead to blood stasis, increasing the risk of thrombus formation, which may embolize into the peripheral arteries.^5^ Recent data show a shift in ALI etiology, from valvular causes in younger populations to embolization from atrial fibrillation or ventricular thrombi in elderly patients.^7^ In the current case, the patient was elderly (65 years old) and was newly diagnosed as having atrial fibrillation with rapid ventricular response alongside CHF and hypertension, placing her at moderate to high thromboembolic risk (CHA_2_DS_2_-VA score of 3). Although no intracardiac thrombus was detected on echocardiography, the presence of biventricular dysfunction likely facilitated blood pooling and thrombus formation, fulfilling Virchow's triad.^8^ Notably, her preadmission history of intermittent left-hand claudication and mild sensory symptoms may reflect underlying chronic arterial insufficiency, further predisposing the limb to ischemic events. Uncontrolled hypertension could also have contributed to vascular vulnerability and occlusion.^9^

Rapid recognition of ALI is crucial, as outcomes are highly time dependent. The patient developed sudden neurological and vascular deficits in the left upper limb, consistent with the classic “6 Ps” (pain, pallor, pulselessness, paresthesia, paralysis, poikilothermia).^7^ Bedside Doppler ultrasonography revealed absent arterial signals in the axillary artery, confirming Rutherford grade IIb ALI—a limb-threatening stage requiring urgent revascularisation.^10^ DSA subsequently confirmed the occlusion and informed therapeutic planning.^1^^,^^7^

Continuous low-dose heparin infusion was also administered to maintain catheter patency. In accordance with current guidelines, systemic anticoagulation with intravenous heparin was initiated promptly to prevent thrombus propagation.^3^^,^^7^ Given the facility's limited infrastructure, a practical hybrid strategy was employed—manual thrombosuction using a Judkins right catheter and 10-mL syringe, followed by CDT with intra-arterial alteplase. Low-dose heparin was infused through the sheath to maintain catheter patency.^7^ Despite the absence of advanced thrombectomy devices, this approach successfully restored perfusion to the palmar arch, as evidenced by completion angiography. No signs of compartment syndrome or reperfusion injury were observed, and fasciotomy was not required.

The patient demonstrated significant clinical improvement and was discharged with optimized GDMT for heart failure, warfarin, and statin therapy for long-term secondary prevention.^6^ This case underscores that even in nontertiary, resource-constrained hospitals, early recognition and a pragmatic, endovascular-first approach can lead to excellent outcomes in ALI, reinforcing the importance of timely decision-making and the effective use of available tools.

## Conclusions

This case highlights the critical importance of early recognition and timely intervention in upper ALI, particularly in patients with underlying cardioembolic risk factors such as atrial fibrillation and heart failure. Despite being managed in a resource-limited, nontertiary hospital, a pragmatic endovascular approach combining manual thrombosuction and CDT led to successful limb salvage. This demonstrates that with prompt clinical decision-making and adherence to evidence-based guidelines, favorable outcomes can be achieved even in settings with limited vascular resources.

**Visual Summary:** Timeline of the Case

Time	Events
Day 1	A 65-year-old woman presented with palpitations and shortness of breath. ECG showed AF-RVR at 140 beats/min. Echocardiography revealed reduced LVEF (30.2%) and RV dysfunction (TAPSE: 8 mm).
Day 3	After stabilization, she developed left upper ALI with absent pulses, delayed CRT, undetectable oxygen saturation, sensory loss, and weakness. DUS and DSA confirmed complete occlusion of the left axillary artery. She was treated with IV heparin, followed by endovascular manual thrombosuction and catheter-directed thrombolysis using alteplase.
Day 4	Postintervention angiography showed restored perfusion to the hand, with improved oxygenation and palpable pulses.
Day 6	The patient was discharged on GDMT for heart failure, warfarin 4 mg, and atorvastatin 40 mg.
Follow-up day 7	Seven-day follow-up showed good recovery without issues.
Follow-up day 30	Thirty-day follow-up indicated sustained improvement and stable condition.

AF-RVR = atrial fibrillation with rapid ventricular response; ALI = acute limb ischemia; CRT = capillary refill time; DUS = Doppler ultrasound; DSA = digital subtraction angiography; ECG = electrocardiogram; GDMT = guideline-directed medical therapy; IV = intravenous; LVEF = left ventricular ejection fraction; RV = right ventricular; TAPSE = tricuspid annular plane systolic excursion.

**Equipment List:** 

Imaging • Doppler ultrasound (Philips Healthcare) • Linear probe (L12-3)
Access • Needle puncture 18 F • Hydrophilic wire 0.035 and 7-F sheath femoral
Manual thrombosuction • Judkins right catheter (JR 3.5/6-F) • Spuit 10 mL syringe

## Funding Support and Author Disclosures

The authors have reported that they have no relationships relevant to the contents of this paper to disclose.
